# Liver-specific *in vivo* base editing of *Angptl3* via AAV delivery efficiently lowers blood lipid levels in mice

**DOI:** 10.1186/s13578-023-01036-0

**Published:** 2023-06-15

**Authors:** Yuanbojiao Zuo, Chen Zhang, Yuan Zhou, Haiwen Li, Weidong Xiao, Roland W. Herzog, Jie Xu, Jifeng Zhang, Y. Eugene Chen, Renzhi Han

**Affiliations:** 1 Herman B Wells Center for Pediatric Research, Indiana University School of Medicine, Indianapolis, IN 46202 USA; 2 Department of Surgery, The Ohio State University, Columbus, OH 43210 USA; 3 Department of Pediatrics, Third Xiangya Hospital of Central South University, Changsha, 410013 Hunan P.R. China; 4 Center for Advanced Models for Translational Sciences and Therapeutics, University of Michigan Medical Center, Ann Arbor, MI USA

**Keywords:** ANGPTL3, Cholesterol, Base editing, Base editor, Cardiovascular disease, CVD, Triglyceride

## Abstract

**Background:**

Gene editing has emerged as an exciting therapeutic development platform for numerous genetic and nongenetic diseases. Targeting lipid-modulating genes such as *angiopoietin-related protein 3* (*ANGPTL3*) with gene editing offers hope for a permanent solution to lower cardiovascular disease risks associated with hypercholesterolemia.

**Results:**

In this study, we developed a hepatocyte-specific base editing therapeutic approach delivered by dual adeno-associated virus (AAV) to enable hepatocyte-specific targeting of *Angptl3* to lower blood lipid levels. Systemic AAV9-mediated delivery of AncBE4max, a cytosine base editor (CBE), targeting mouse *Angptl3* resulted in the installation of a premature stop codon in *Angptl3* with an average efficiency of 63.3 ± 2.3% in the bulk liver tissue. A near-complete knockout of the ANGPTL3 protein in the circulation were observed within 2–4 weeks following AAV administration. Furthermore, the serum levels of triglyceride (TG) and total cholesterol (TC) were decreased by approximately 58% and 61%, respectively, at 4 weeks after treatment.

**Conclusions:**

These results highlight the promise of liver-targeted *Angptl3* base editing for blood lipid control.

**Supplementary Information:**

The online version contains supplementary material available at 10.1186/s13578-023-01036-0.

## Introduction

Elevated levels of low-density lipoprotein cholesterol (LDL-C) and very-low-density lipoprotein cholesterol (VLDL-C) play dominant roles among all the different mechanisms involved in cardiovascular disease (CVD) [1, 2]. The inhibitors of 3-hydroxy-3-methylglutaryl coenzyme A (HMG-CoA) reductase (statins) reduce cardiovascular risk by approximately 50–60% [3, 4]. In recent trials, combination therapies of statins and non-statin agents such as proprotein convertase subtilisin/kexin type 9 (PCSK9) inhibitors (PCSK9i) are proven effective in promoting coronary atherosclerosis regression in patients with moderate-to-high CVD risk [5–9]. However, a substantial number of patients who lack sufficient functional LDL receptor (LDLR) and are unable to reach sufficiently low levels of LDL-C (≤ 70 mg/dl in non-familial hypercholesterolemia), are drug intolerant, or do not meet the U.S. Food and Drug Administration (FDA) and the 2017 clinical American College of Cardiology Expert consensus guidelines [1] for combination therapies. New avenues of treatment are needed for these patients.

Angiopoietin-related proteins are important regulators of lipoprotein metabolism. ANGPTL3 is an endogenous inhibitor of lipoprotein lipase (LPL), which is the main enzyme involved in the hydrolysis of TG-rich lipoproteins and LDL for their clearance from the circulation [10]. Population studies have shown that individuals with the loss-of-function (LoF) variants in *ANGPTL3* had significantly lower TG levels and an over 40% lower risk of cardiovascular events [11–13]. Monoclonal antibody, antisense oligonucleotide and gene editing therapies have been pursued to inactivate *ANGPTL3* as a therapeutic target. Evinacumab, a monoclonal anti-ANGPTL3 antibody developed by Regeneron, received FDA approval in 2021 [14]. Mice treated with antisense oligonucleotides (ASOs) targeting *Angptl3* messenger RNA had decreased serum TG, and LDL-C levels and similar findings were reported in clinical trial [15, 16]. Since the effect of ANGPTL3 inhibition on lipid levels is independent of LDLR function [17], ANGPTL3 inhibitors may benefit hypercholesterolemia patients who do not respond sufficiently to statin or PCSK9 therapy, and further reduce CVD risks for patients who are already on statin or PCSK9 drugs.

In contrast to monoclonal antibodies and ASOs that require repeated administration, *in vivo* gene editing holds the potential of “one-shot-cure” by introducing permanent LoF mutations in the target genes. Qiu et al. reported that lipid nanoparticle-mediated delivery of Cas9 mRNA and *Angptl3*-targeting gRNA profoundly reduced serum LDL-C and TG levels in C57BL/6 mice [18]. However, Cas9-induced DNA double-strand breaks (DSBs) and undesired off-target mutations raised safety concerns. CRISPR-derived base editors, on the other hand, can install point mutations in the genome without introducing DSBs [19, 20]. Chadwick et al. [21] employed adenovirus to deliver base editor targeting *Angptl3* in mice and observed reduced serum levels of TG and TC in the treated animals.

Here we explored the feasibility of AAV-delivered base editor targeting *Angptl3*. We demonstrated that base editing can induce efficient editing of mouse *Angptl3* in Neuro-2a (N2a) cells. We then employed the Gp41-1 intein split approach [22] to package AncBE4max [23] under the control of the hepatocyte-specific promoter human alpha-1-antitrypsin (hAAT) [24–26] and the gRNA targeting mouse *Angptl3* into AAV9. Systemic delivery of AAV9-hAAT-AncBE4/*Angptl3* conferred efficient editing of *Angptl3* in liver and resulted in significantly reduced serum levels of ANGPTL3, TG and TC. These results highlight the therapeutic potential of *in vivo ANGPTL3* base editing for plasma lipid control.

## Results

### *In vitro* validation of *Angptl3*-targeting gRNAs with ABE or CBE

**Fig. 1:**
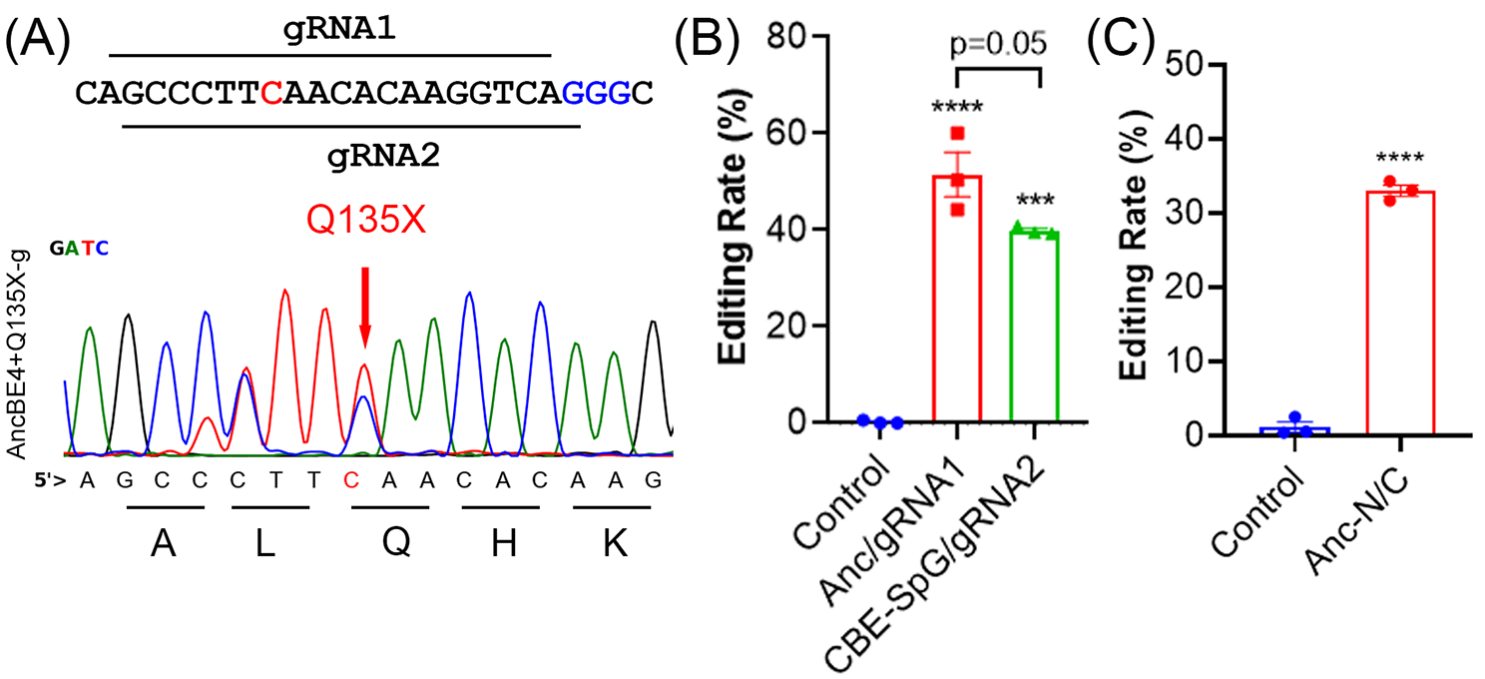
*In vitro* validation of *Angptl3* gRNAs to introduce a premature stop codon with CBE. (**A**) Schematic showing two overlapping *Angptl3* gRNAs (gRNA1 and gRNA2) with different PAM-targeting CBEs (AncBE4max or CBE-SpG). The targeted cytosine is colored in red and the PAM sequences are colored in blue. (**B**) Comparison of the editing efficiency of gRNA1 and gRNA2 at mouse *Angptl3* locus. ****p < 0.0001, ***p < 0.001; one-way ANOVA with Turkey’s multiple comparisons test. (**C**) Measurement of base editing efficiency induced by *Angptl3*- gRNA1 when combined with N- and C-terminal halves of AncBE4max. ****p < 0.001; two-tailed unpaired *t* test

To induce LoF mutation, we designed two overlapping gRNAs to install a premature stop codon (Q135X) into the coding sequence of mouse *Angptl3* using AncBE4max [23] (Fig. 1A). At 72 h after transfection of N2a cells with the gRNA and base editor, the gRNA1 and AncBE4max combination induced 51.5 ± 4.6% conversion of C to T at the Q135 codon, while the efficiency of gRNA2 and CBE-SpG was 39.8 ± 0.5% (Fig. 1B). Given the higher editing efficiency of gRNA1 and AncBE4max, we chose this combination for our *in vivo* studies.

As the size of AncBE4max together with the regulatory sequences is beyond the packaging capacity of AAV, we took the intein split strategy as we previously reported [22] to split AncBE4max into two halves and tested in N2a cells for editing *Angptl3*. Co-transfection of N2a cells with AncBE4max-N and -C vectors resulted in robust editing of *Angptl3* to mutate the Q135 codon (Fig. 1C).

### *In vivo* performance of AAV9-hAAT-AncBE4/*Angptl3* in mice

**Fig. 2:**
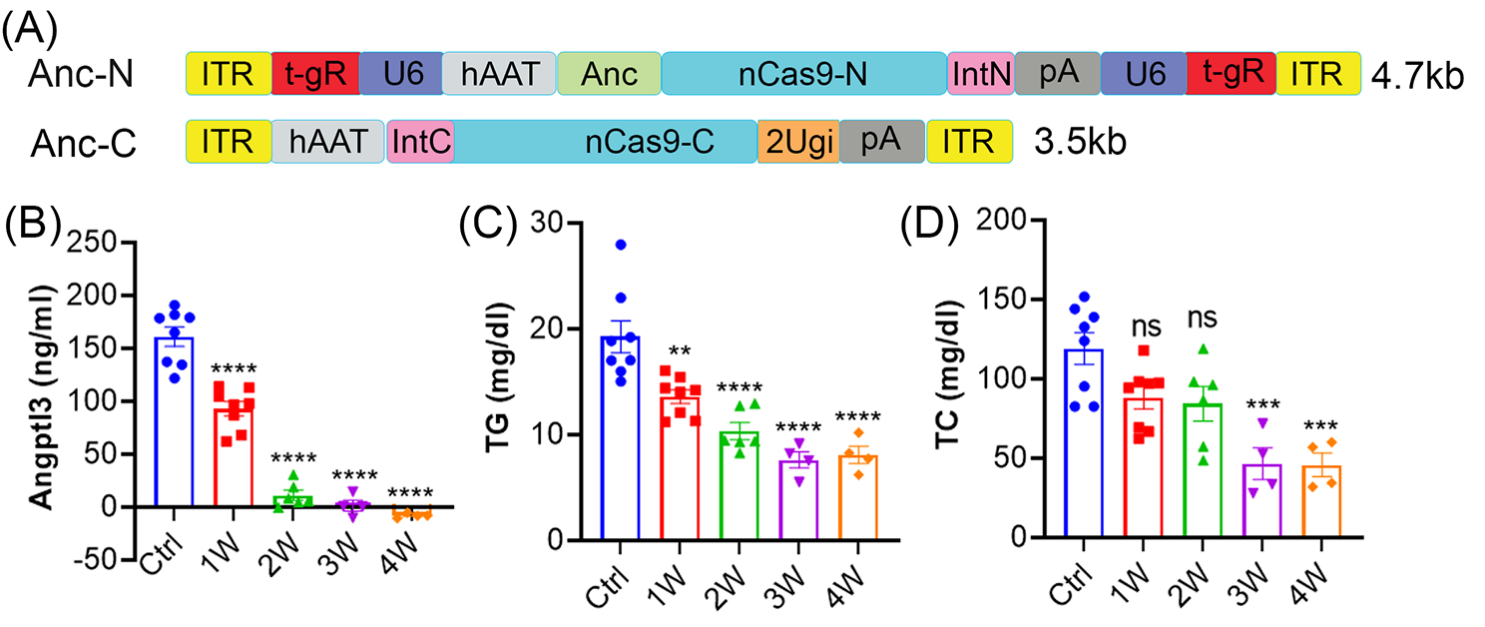
*In vivo* performance of AAV9-hAAT-Anc/*Angptl3* in mice. (**A**) Schematic showing AAV9-hAAT-Anc/*Angptl3* N- and C-terminal halves design. (**B**) Measurements of the serum levels of ANGPTL3 protein. (C, D) Measurements of serum TG (**C**) and TC (D) in mice at 1–4 weeks after AAV9-hAAT-Anc/*Angptl3* administration. The control (Ctrl) mice received no treatments. ns, not significant; **p < 0.01, ***p < 0.001 and ****p < 0.0001; one-way ANOVA with Turkey’s multiple comparisons test

As *Angptl3* is a hepatocyte-specific gene [27, 28], we used the hepatocyte-specific promoter, human alpha-1-antitrypsin [24–26], to drive the expression of AncBE4max. The N- and C-terminal halves of the hAAT-AncBE4max together with the U6-driven gRNA cassette were packaged into two AAV9 vectors (Fig. 2A). The AAV9-hAAT-AncBE4/*Angptl3* (a total of 2 × 10^14^ vg/kg, 1:1 of the N- and C-terminal halves) was administered into eight male C57BL/6J mice (6 weeks old) through tail vein injection. Two mice were sacrificed at one week and another two at two weeks after injection for verification of gene editing (Supplementary Fig. 1).

Blood samples were collected weekly to assess the targeted protein and circulating lipid levels. All AAV9-injected animals exhibited dramatically decreased ANGPTL3 protein levels starting at one week after treatment (after: 92.9 ± 6.8 ng/ml vs. before: 161.2 ± 9.2ng/ml) and achieved nearly complete protein knockdown at 2–4 weeks (Fig. 2B). Concordantly, AAV9-hAAT-AncBE4/*Angptl3* also dramatically decreased the TG levels from 19.3 ± 1.5 mg/dl to 8.1 ± 0.8 mg/dl in all animals at 4 weeks after treatment (Fig. 2C). As disrupted expression of ANGPTL3 protein are known to reduce both cholesterol and triglycerides [29], we measured serum TC levels, which were also significantly reduced by AAV9-hAAT-AncBE4/*Angptl3* treatment starting from 3 weeks post administration (Fig. 2D).

**Fig. 3:**
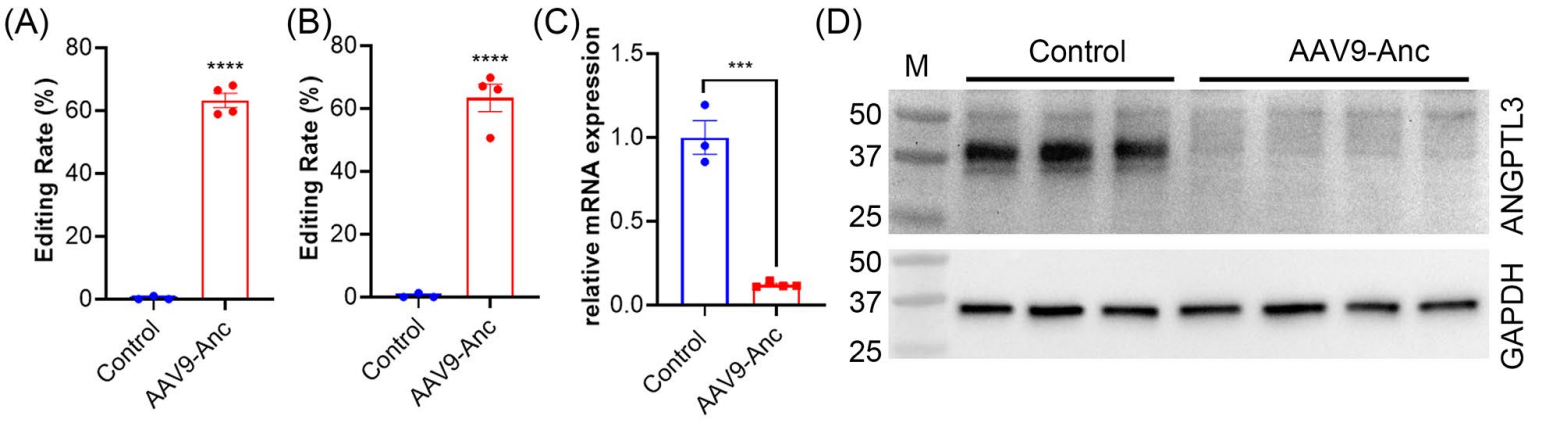
Base editing outcomes of *Angptl3* in the liver tissues of the mice. (**A, B**) Quantification of editing efficiency at the genomic DNA (**A**) and the transcript (**B**) levels by BEAT analysis of Sanger sequencing data. AAV9-Anc, AAV9-hAAT-Anc/*Angptl3* treated group. ****p < 0.0001; two-tailed unpaired *t* test. (**C**) qRT-PCR analysis of *Angptl3* expression in the liver tissues of mice treated with or without AAV9-hAAT-Anc/*Angptl3*. ***p < 0.001; two-tailed unpaired *t* test. (**D**) Western blot analysis of ANGPTL3 in the mouse liver tissues treated with or without AAV9-hAAT-Anc/*Angptl3* (60 µg per lane). GAPDH was probed as a loading control

At 8 weeks after AAV administration, liver tissues were harvested from the mice for gene editing analysis. Sequencing the PCR amplicons of genomic DNA isolated from the bulk mouse liver tissues showed that AAV9-hAAT-AncBE4/*Angptl3* treatment led to an editing efficiency of 63.3 ± 2.3% at *Angptl3* (Fig. 3A). Sequencing of the *Angptl3* transcripts showed similar results (Fig. 3B). To examine if the gene editing reduced the transcript expression levels of *Angptl3*, we performed quantitative reverse transcription-polymerase chain reaction (qRT-PCR) on the liver tissues collected from the AAV9 treated and control mice. As shown in Fig. 3C, the transcript expression of *Angptl3* was dramatically reduced by 88.0 ± 0.8% in AAV9 treated mice. Consistently, Western blot showed that the ANGPTL3 protein in the liver tissues of AAV9-treated animals was reduced to undetectable levels (Fig. 3D).

To assess whether hAAT-driven AncBE4 expression confers liver specificity, we measured the editing rate at the *Angptl3* locus in off-target tissues (heart, kidney, skeletal muscle) collected from the AAV9 treated animals. Sanger sequencing analysis showed un-detectable editing in any of these tissues (Supplementary Figs. 2–4).

### Evaluation of liver toxicity and off-target activities

**Fig. 4:**
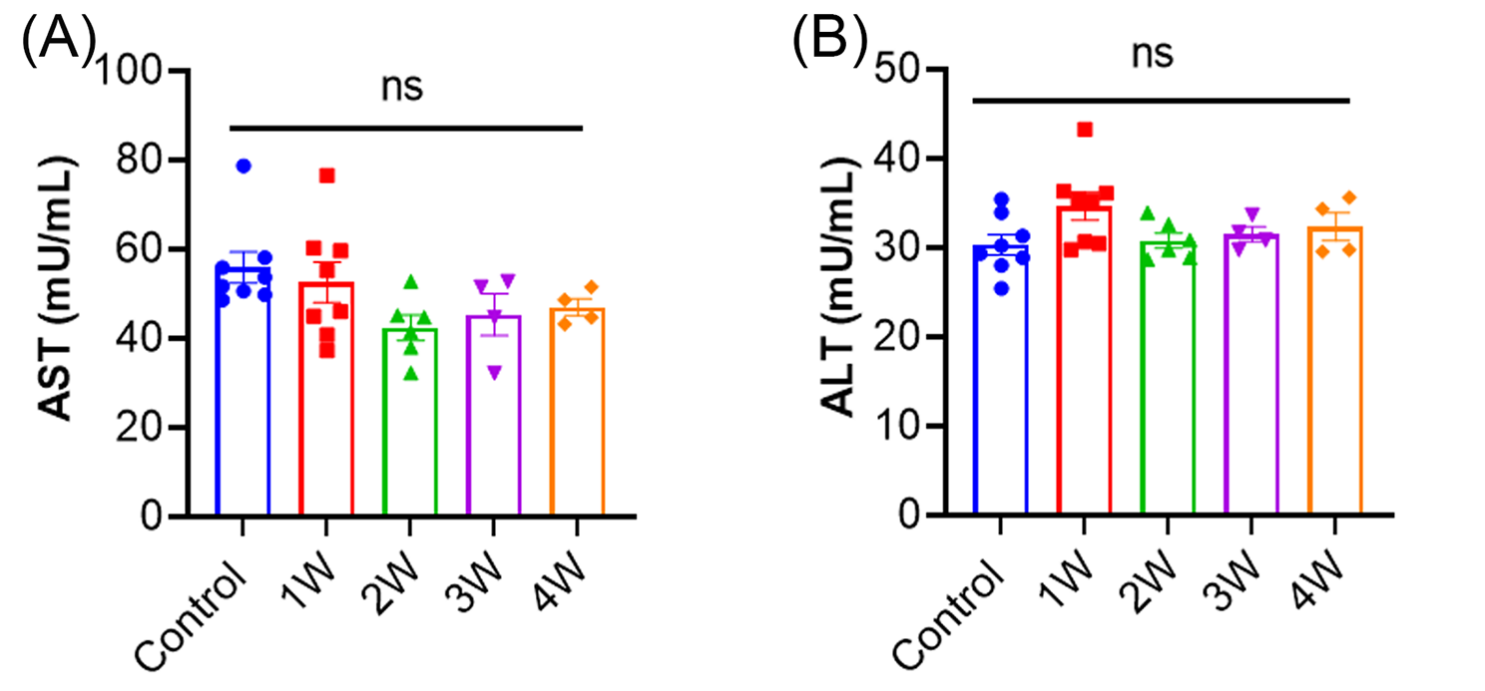
Liver toxicity analysis in mice treated with AAV9-hAAT-Anc/*Angptl3*. (**A, B**) Measurement of serum AST and ALT levels in mice at first four weeks after treatment with AAV9-hAAT-Anc/*Angptl3*. ns, not significant; one-way ANOVA with Turkey’s multiple comparisons tests

To examine if AAV9-delivered AncBE4 induced liver toxicity, we first measured the serum levels of aspartate aminotransferase (AST) and alanine aminotransferase (ALT). There were no significant changes in either AST or ALT levels in the treated animals at 1 to 4 weeks after AAV9 delivery (Fig. 4A, B). We also performed histological examination of the mouse liver tissues by hematoxylin and eosin (H&E) staining, which showed no signs of inflammation such as aggregates of lymphocytes or macrophages were detected (Supplementary Fig. 5A). Immunofluorescence staining with the anti-CD3 antibody did not detect increased CD3 + T lymphocyte infiltration (Supplementary Fig. 5B). These results suggest that AAV9-hAAT-Anc/*Angptl3* treatment exhibited no detectable liver toxicity or T cell infiltration.

**Fig. 5:**
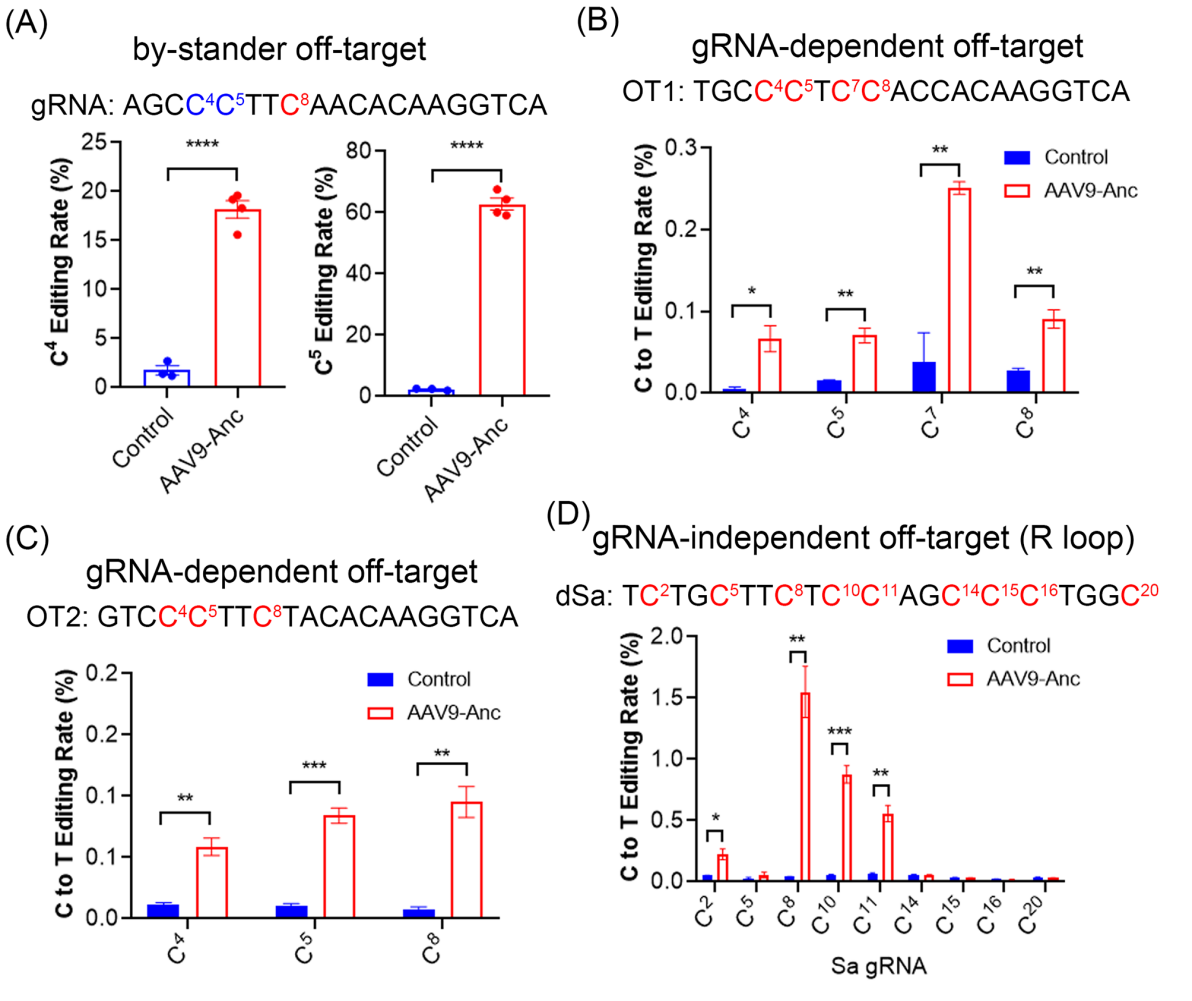
*In vivo* and *in vitro* OT activity analysis. (**A**) BEAT quantification of the bystander C4 and C5 editing in mice treated with or without AAV9. ****p < 0.0001; two-tailed unpaired *t* test. (**B, C**) Targeted deep sequencing of the genomic DNA PCR amplicons of the OT1 (B) and OT2 (**C**) from N2a cells transfected with or without *Angptl3*-gRNA1 combined with N- and C-terminal halves of AncBE4max. *p < 0.05; **p < 0.01; ***p < 0.001; two-tailed unpaired *t* test. (**D**) Targeted deep sequencing to quantify the gRNA-independent off-target editing using orthogonal R-loop assay. *p < 0.05; **p < 0.01; ***p < 0.001; two-tailed unpaired *t* test

One concern over *in vivo* base editing is the potential off-target (OT) activities such as bystander editing, gRNA mismatch tolerance and gRNA-independent editing. There are several cytosines (e.g., C4 and C5) at the *Angptl3* target site within the targeting window of AncBE4max. As expected, we found that both cytosines at position 4 and 5 were highly edited in the liver tissues from mice treated with AAV9-hAAT-Anc/*Angptl3* (Fig. 5A). Prediction by Cas-OFFinder [30] showed that a total of twelve sites have three mismatches. As Cas9-based base editors are less tolerant to the mismatches in the last 12 positions proximal to the protospacer adjacent motif (PAM) sequence [31], we selected two OT sites that have no mismatches in the last 12 positions: OT1 (located on chromosome 1) and OT2 (located on chromosome 16). Targeted deep sequencing of the genomic DNA amplicons from N2a cells showed that there were significant but low levels of editing activities at both OT1 and OT2 sites (Fig. 5B&C). Finally, to detect gRNA-independent off-target editing activities, we performed the orthogonal R-loop assay [32], in which a gRNA and dead SaCas9 (dSaCas9) were used to induce a R-loop formation and the editing of this open loop by AncBE4max without its cognate gRNA was examined by targeted deep sequencing. Consistent with previous reports [32, 33], AncBE4max induced low but significant gRNA-independent editing along the R loop (Fig. 5D).

## Discussion

In this study, we utilized a dual-AAV system to deliver AncBE4max with a heptaocyte-specific promoter targeting mouse *Angptl3* in order to achieve lipid lowering. Systemic AAV9-mediated delivery of AncBE4max induced 63.3 ± 2.3% genome editing at the *Angptl3* locus to install Q135X premature stop codon. As a result of *Angptl3* base editing, we observed dramatically decreased ANGPTL3 protein levels starting from one week after injection, which became nearly undetectable at 2–4 weeks. Moreover, *in vivo* base editing of *Angptl3* significantly lowered blood levels of TG and TC. Thus, this dual AAV-delivered AncBE4max enables efficient hepatocyte-specific gene editing of *Angptl3*, which could be developed as a potential therapy for reducing atherosclerotic cardiovascular risk.

The base editing shows great promise in correcting numerous inherited disorders [19, 20, 34]. However, the large size of base editors makes the choices of carrier vehicle for safe and efficient delivery very limited. Our previous study reported a dual trans-splicing AAV approach to deliver ABE using split intein Gp41-1, achieving robust editing efficiency to correct a Duchenne muscular dystrophy mutation in adult mice [22]. Similar approaches have been previously utilized to deliver CBE [35, 36]. In the current study, we adopted this Gp41-1 intein split approach to deliver AncBE4max to achieve tissue-specific gene editing. To balance the size of N- and C- terminal halves of AncBE4max, we chose the amino acid position 573 and 574 of the Cas9 nickase as the splitting site because previous studies showed that 573/574 split Cas9 exhibited near the full-length Cas9 activity [37]. The cell specificity was achieved with the combination of a highly efficient AAV serotype and a hepatocyte-specific promoter to drive the AncBE4max expression. This approach enabled efficient hepatocyte-specific editing of *Angptl3* and could be broadly implemented to achieve other tissue-specific base editing.

Interestingly, our measured editing rates at both the DNA and RNA levels were about 63% while the ANGPTL3 protein showed almost a complete disruption. This discrepancy is likely due to an under-estimation of the editing rates at both the DNA and RNA levels for the following reasons. Liver tissues contain only ~ 52–70% of hepatocytes [38, 39] and the other cell types were unlikely to be edited due to the utilization of liver specific hAAT promoter to drive the base editor expression. Our qRT-PCR data showed that the Angptl3 transcript level in the edited livers was greatly reduced to only 12% of control livers, suggesting that the majority of the edited transcripts in the edited livers had been degraded likely due to the nonsense-mediated decay. However, from the WT transcript leftover in the edited samples (12% x (1-63.4%) = 4.4%), we can estimate that the editing rate at the transcript level is about 95.6%, matching what we observed at the protein level as detected by Western blot and ELISA.

Although highly efficient, the AAV delivery could cause serious adverse events as reported in several recent clinical trials [40–43]. Prolonged expression of the transgene delivered in AAV vectors can be problematic when the transgene expression is needed for only a very limited duration as in the case of gene editing. The continued expression of base editors can potentially lead to accumulation of unwanted off-target editing events (see Fig. 5) and elicit undesired host immune responses against the edited cells [22, 44]. Further efforts would be required to develop novel approaches to offer temporal control of the gene editing reagents. Alternatively, transient delivery of base editors in the forms of ribonucleoprotein (RNP) [45–47] or mRNA [18, 48, 49] instead of DNA using non-viral approaches can bypass the size limitation, maintain the high editing efficiency and lower the OT activities. Lipid nanoparticle (LNP), formed by lipids and polyethylene glycol (PEG) encapsulating transgene materials, has been developed as a leading non-viral delivery method in gene therapy. Delivery of chemically modified ABE mRNA and gRNA targeting *Pcsk9* via LNP achieved over 60% editing in mouse liver and 95% knockdown in serum PCSK9 protein levels [50]. Moreover, ABE mRNA was rapidly degraded within 12 h after injection and no DNA off-target editing was detected in treated mice. Recently, Banskota et al. [47] engineered viral-like particles (VLPs) to encapsulate base editor protein and *Pcsk9*-targeting gRNA. Systemic injections of the particles into mice achieved 63% editing in liver, resulting in a 78% reduction in serum PCSK9 protein levels in treated mice. However, the tissue specificity, scale-up feasibility and storage stability of such LNP- or VLP-packaged mRNA or RNP delivery approaches remain to be addressed in future studies.

## Materials and methods

### Ethics statement

The C57BL/6J mice purchased from the Jackson Laboratory and housed at The Ohio State University Laboratory Animal Resources in accordance with animal use guidelines. All the experimental procedures were approved by the Institutional Animal Care and Use Committee of the Ohio State University. All mice were maintained under standard conditions of constant temperature (72 ± 4 °F), humidity (relative, 30–70%), in a specific pathogen-free facility and exposed to a 12-h light/dark cycle.

### Generation of AAV particles and administration into mice

The AAV9 vectors were produced and purified by Andelyn Biosciences [51]. All AAV9 vectors were titered using digital droplet PCR. Titers are expressed as DNase resistant particles in vector genome per ml (vg/ml): 2.7 × 10^13^ vg/ml for AAV9-hAAT-AncBE4-N and 2.3 × 10^13^ vg/ml for AAV9-hAAT-AncBE4-C. AAV9 particles (total: 2 × 10^14^ vg/kg, 1:1 of N and C) were systemically administered into a total of eight C57BL/6J mice at 6 weeks of age through tail vein injection as described previously [22]. Two mice were sacrificed at 1 week and two additional mice at 2 weeks after AAV administration to examine the gene editing efficiency. Blood samples were collected via facial vein before and weekly after AAV9 injection for four weeks. At 8 weeks after AAV9 injection, all the rest animals were sacrificed and liver tissues were harvested for gene editing analysis.

### Plasmid construction

The CBE plasmids including pCAG-CBE4max-SpG-P2A-EGFP [52] (Addgene #139,998), pCMV_AncBE4max [23] (Addgene #112,094) and pAAV-pCMV-dSaCas9-VP64-pU6-sgRNA (Addgene #158,990) were obtained from Addgene (Watertown, MA). All gRNA oligos were annealed and cloned into pLenti-ogRNA via BsmBI site. The hAAT promoter sequence was PCR amplified from the genomic DNA of HEK293 cells. The hAAT promoter, Gp41-1 intein split base editor fragments, and the human U6-driven gRNA fragment, were PCR amplified and inserted into the AAV transfer plasmids pZC0031 and pZC0033 as described previously [22].

### Cell culture and transfection

N2a cells were maintained in Dulbecco’s Modified Eagle Medium (DMEM) (Fisher Scientific, 11-965-092) with 10% FBS and 1%100X penicillin-streptomycin (Thermo Fisher Scientific, 15,140,122) in 10 cm^2^ dish. Medium were replaced every 2 days or as needed. Cells were seeded in 6-well plates and transfected with 2 µg plasmids when cell density reached ~ 70% confluency. For gRNA and base editor co-transfection, the ratio of the two plasmids was 1:1. The transfection was conducted by X-tremeGENE HP DNA trasfection reagent (MilliporeSigma, 6,366,546,001) according to manufacturer’s protocol.

### Genomic DNA and total RNA extraction, PCR and Sanger sequencing

Genomic DNA extraction was extracted using DNA lysis buffer (100 mM Tris-Cl pH 8.0, 50 mM EDTA and 1% SDS) with proteinase K according to manufacturer’s instruction. Total RNA was extracted using Quick-RNA MiniPrep Kit (ZYMO Research, R1055) and RT-PCR was conducted using RevertAid RT Reverse Transcription Kit (Thermo Fisher Scientific, K1691) with 100 ng RNA as template. One µl cDNA or 100 ng genomic DNA was used as template in regular PCR reactions using GoTaq Master Mix (Promega, M7122). The PCR products were purified by Wizard SV Gel and PCR Clean-Up System (Promega, A9285) according to manufacturer’s protocol. Purified products were sent for Sanger sequencing at the Genomics Shared Resource of the Ohio State University Comprehensive Cancer Center and analyzed by the BEAT program [53]. Quantitative RT-PCR (qRT-PCR) was performed using PowerUp™ SYBR™ Green Master Mix (Thermo Fisher Scientific, A25741) in QuantStudio™ 5 Real-Time PCR System (Thermo Fisher Scientific, A34322) using 1 µg cDNA and normalized to glyceraldehyde 3-phosphate dehydrogenase (*Gapdh*).

### Orthogonal R-loop assay and targeted deep sequencing

The gRNA-independent off-target editing activities were examined by the orthogonal R-loop assay [32]. Briefly, HEK239 cells were transfected with catalytically dead SaCas9 (dSaCas9) and a cognate gRNA (TCTGCTTCTCCAGCCCTGGC, PAM: CTGGGT) as well as AncBE4max without its cognate gRNA. At 72 h after transfection, the genomic DNA was extracted, and the dSaCas9 gRNA target site was amplified. The amplicons were subjected to next-generation sequencing (NGS) using a MiSeq nano-scale flow cell (Paired-end 300 base-pair reads) at The Genomics Services Laboratory of Nationwide Children’s Hospital. Sequencing data were analyzed using CRISPResso2 [54].

The Angptl3 off-target loci (OT1: TGCCCTCCACCACAAGGTCA PAM: GGG; OT2: GTCCCTTCTACACAAGGTCA PAM: GGG) were first amplified by genomic DNA PCR using site-specific primers (OT1-F: CGTGTGCTTCCACAGTTCAT; OT1-R: CAATCCAGGCCTTCAGACAT; OT2-F: CTGTCCCAGCTCCTCTTTTG; OT2-R: CAATGTTTTCCTTGGCTGGT). The PCR products were purified by Wizard SV Gel and PCR Clean-Up System (Promega, A9285) according to manufacturer’s protocol. Purified products were diluted to equimolarity at 20 ng/µl and submitted to GeneWiz (Azenta Life Sciences) for NGS (Amplicon-EZ: Illumina MiSeq, 2 × 250 bp sequencing, paired end). Sequencing data were analyzed using CRISPResso2 [54].

### Western blot analysis

Liver tissues from mice treated with or without AAV9-hAAT-AncBE4/*Angptl3* were lysed with cold RIPA buffer supplemented with 10x protease inhibitor. Protein samples were separated using 4–15% SDS-PAGE gel (Bio-Rad, 17,000,927) and transferred onto 0.45 µM polyvinylidene difluoride membrane. The goat polyclonal anti-ANGPTL3 (R&D Systems, AF136, 1:1500) and rabbit monoclonal anti-GAPDH (Cell Signaling Technology, 2118 S, 1:2000) antibodies were used for immunoblotting analysis as primary antibodies. HRP conjugated goat anti-rabbit (Cell Signaling Technology, 7074 S, 1:4000) and chicken anti-goat IgG (R&D Systems, HAF019, 1:2000) were used as secondary antibodies. The membranes were developed using ECL Western blotting substrate (Pierce Biotechnology, Rockford, IL) and scanned by ChemiDoc XRS + system (BioRad, Hercules, CA).

### Immunofluorescence staining

Liver tissues were harvested from mice treated with or without AAV9-hAAT-Anc/*Angptl3* at 8 weeks after AAV9 injection. Liver tissues were embedded in optimal cutting temperature (OCT, Sakura Finetek, Netherlands) compound and snap-frozen in cold isopentane for cryosectioning. The tissues were stored at − 80 °C. Frozen cryosections (7 μm) were fixed with 4% paraformaldehyde for 15 min at room temperature. After washing with PBS, the slides were blocked with 3% BSA for 1 h. The slides were incubated with primary antibodies against CD3 (Abcam, ab5690, 1:100) at room temperature for 1 h, followed by 3 times PBS wash and incubation with secondary antibodies Alexa Fluor 488 Donkey anti-Rabbit IgG (Thermo Fisher Scientific, A21206, 1:400) for 1 h at room temperature. The slides were then washed 3 times with PBS and sealed with VECTASHIELD Antifade Mounting Medium with DAPI (Vector Laboratory, Burlingame, CA). All images were taken under a Nikon Ti-E fluorescence microscope (magnification 200x) (Nikon, Melville, NY).

### H&E staining

Ten microns of frozen sections were fixed in 10% formaldehyde for 5 min at room temperature and then proceeded to the standard protocol of H&E staining as described previously [55]. All images were taken under a Nikon Ti-E fluorescence microscope, magnification x200.

### Quantification of plasma lipid profile

Blood samples collected from the animals were allowed for clotting at room temperature for 10 min. Serum was isolated by centrifugation at 5000 rpm for 10 min and stored at -80 °C in small aliquots. Determination of TG and TC was performed using the triglyceride and cholesterol assay kit (Abcam, ab65336 and ab65390, respectively) according to the manufacture’s instruction.

### ELISA measurement of serum ANGPTL3

The serum ANGPTL3 protein levels were determined by using the mouse Angiopoietin-like 3 quantikine ELISA Kit (R&D systems, MANL30) according to the manufacture’s instruction.

### Statistical analysis

The data are expressed as the mean ± SEM, analyzed by using GraphPad Prism v.8.0.1 (GraphPad Software). Statistical differences were determined by two-tailed unpaired Student’s *t* test for two groups and one-way ANOVA with Turkey’s post tests for multiple group comparisons. A *p* value < 0.05 was considered as significant.

## Electronic supplementary material

Below is the link to the electronic supplementary material.

Supplementary Material 1

## Data Availability

All relevant data supporting the key findings of this study are available within the article and its Supplementary Information files or from the corresponding author upon reasonable request.

## Abbreviations

AAV: adeno-associated virus
ABE: adenine base editor
ALT: alanine aminotransferase
ANGPTL3: angiopoietin-like 3
ASOs: antisense oligonucleotides
AST: aspartate aminotransferase
CBE: cytosine base editor
CVD: cardiovascular disease
DAPI: 4’,6-diamidino-2-phenylindole
DMEM: Dulbecco’s Modified Eagle Medium
DSBs: double-strand breaks
FDA: Food and Drug Administration
*Gapdh*: glyceraldehyde 3-phosphate dehydrogenase
hAAT: human alpha-1-antitrypsin
H&E: hematoxylin and eosin
HMG-CoA: 3-hydroxy-3-methylglutaryl coenzyme A
LDL-C: low-density lipoprotein cholesterol
LDLR: low-density lipoprotein receptor
LNP: Lipid nanoparticle
LoF: loss-of-function
LPL: lipoprotein lipase
N2a: Neuro-2a
OT: off-target
PAM: protospacer adjacent motif
PCSK9: Proprotein convertase subtilisin/kexin type 9
PEG: polyethylene glycol
RNP: ribonucleoprotein
qRT-PCR: quantitative reverse transcription-polymerase chain reaction
TC: total cholesterol
TG: triglyceride
VLDL-C: very-low-density lipoprotein cholesterol
VLPs: viral-like particles
