# Supplementary material for: Liver-specific in vivo base editing of Angptl3 via AAV delivery efficiently lowers blood lipid levels in mice
Source: Cell Biosci. 2023 Jun 15;13:109. doi: 10.1186/s13578-023-01036-0 (PMC10273718; doi:10.1186/s13578-023-01036-0)
Supplement: Supplementary file 1 — Supplementary Material 1 [file 13578_2023_1036_MOESM1_ESM.pdf]

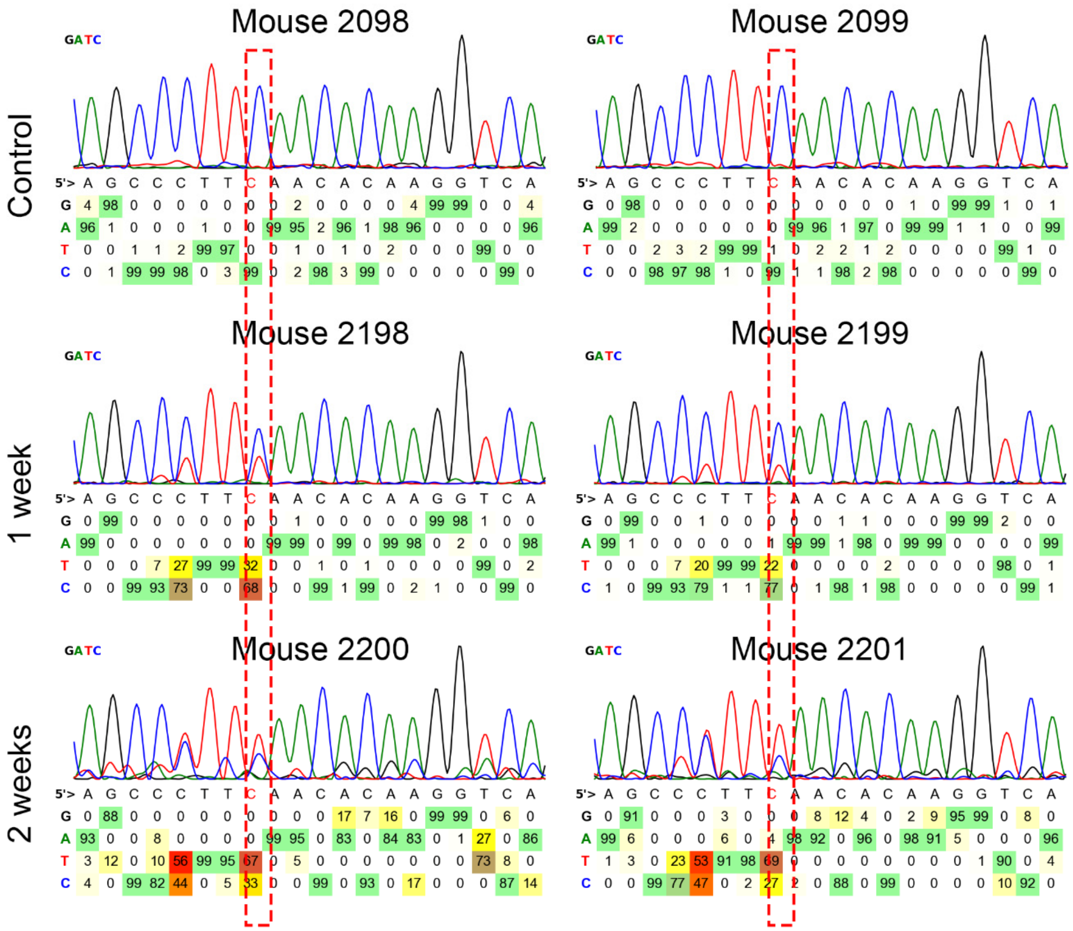

Supplement Figure 1. **Sequencing results of liver genomic DNA PCR amplicons from mice treated with or without AAV9.** Mouse 2098 and 2099 received no treatment and were used as negative control; mouse 2198 and 2199 were sacrificed at 1 week after AAV9 injection; mouse 2200 and 2201 were sacrificed at 2 weeks after AAV9 injection**.**

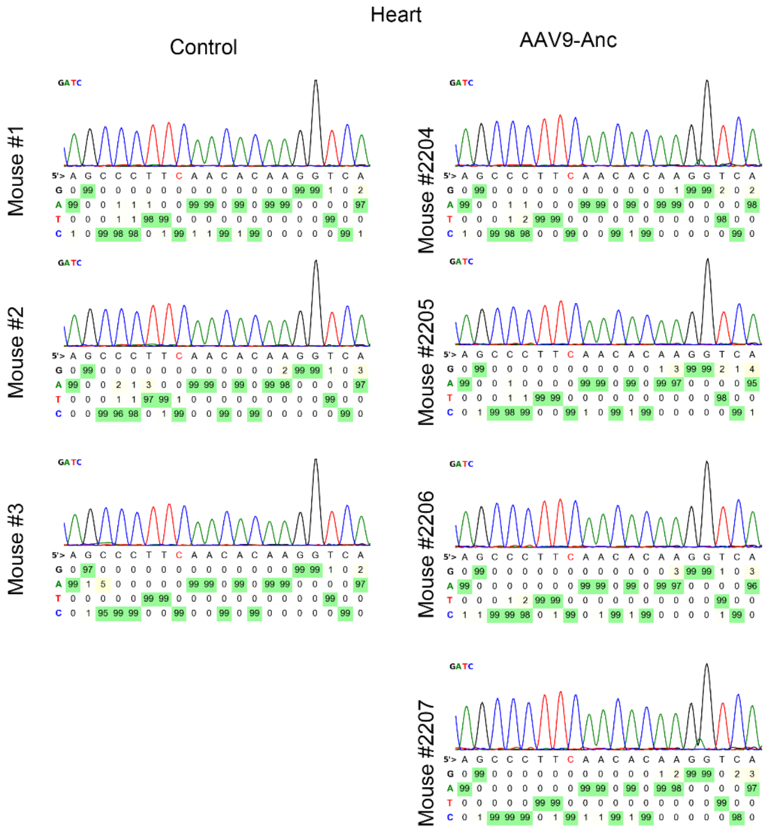

Supplement Figure 2. Sequencing results of heart genomic DNA PCR amplicons from mice treated with or without AAV9. Mouse #1-3 received no treatment and were used as negative control; Mouse #2204-2007 were sacrificed at 8 weeks after receiving AAV9-hAAT-Anc/*Angptl3* treatment.

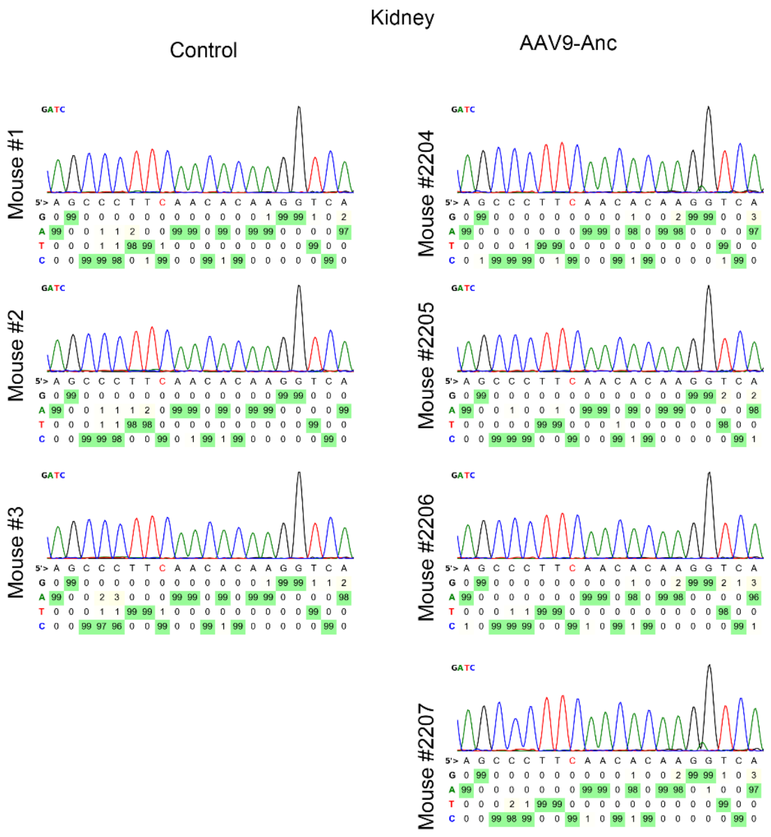

Supplement Figure 3. Sequencing results of kidney genomic DNA PCR amplicons from mice treated with or without AAV9. Mouse #1-3 received no treatment and were used as negative control; Mouse #2204-2007 were sacrificed at 8 weeks after receiving AAV9-hAAT-Anc/*Angptl3* treatment.

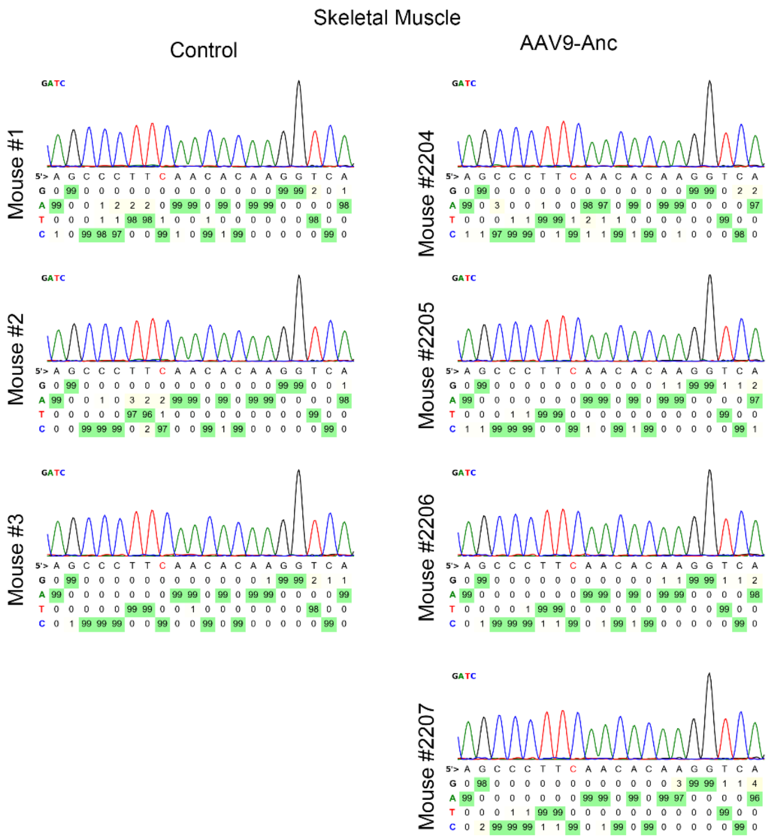

Supplement Figure 4. Sequencing results of skeletal muscle (quadriceps) genomic DNA PCR amplicons from mice treated with or without AAV9. Mouse #1-3 received no treatment and were used as negative control; Mouse #2204-2007 were sacrificed at 8 weeks after receiving AAV9-hAAT-Anc/*Angptl3* treatment.

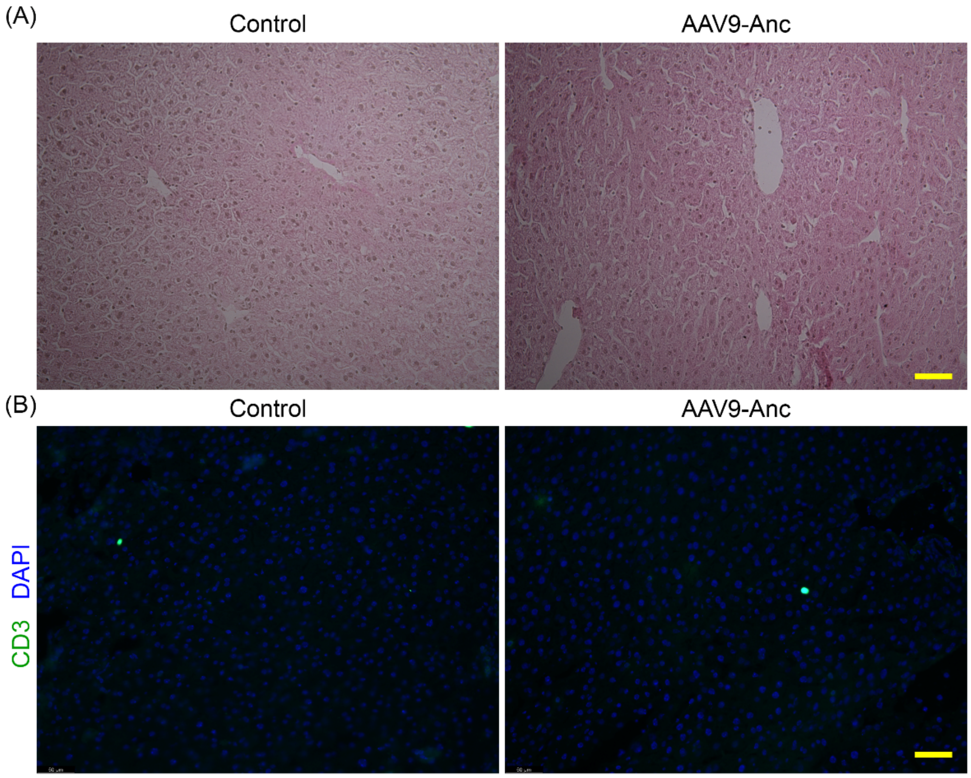

Supplement Figure 5. Immunofluorescence staining of CD3 T cells in liver tissue sections. (A, B) Representative H&E staining (A) and CD3 immunofluorescence staining (B, green) images of mouse liver sections from control and AAV9-hAAT-Anc/*Angptl3* treated mice. 4',6-diamidino-2-phenylindole (DAPI) was used to visualize nucleus. Scale bar: 50 μm.
